# Exploring allosteric efflux pump inhibitors and the role of bacterial membrane potential in modulating drug resistance

**DOI:** 10.3389/fmicb.2026.1802731

**Published:** 2026-04-23

**Authors:** Ajay Pratap Singh Hada, Khem Raj Meena, Jitendra, Gajendra Singh, Hemant Arya, Arvind Kumar

**Affiliations:** 1 Department of Biotechnology, School of Life Sciences, Central University of Rajasthan, Ajmer, India; 2 Department of Biotechnology, Maharaja Surajmal Brij University, Bharatpur, India

**Keywords:** allosteric inhibition, efflux pumps, MDR, membrane potential, pathogens

## Abstract

Multi-drug resistance (MDR) bacteria pose a significant global health challenge, primarily driven by the activity of multi-drug efflux pumps and their intimate coupling to bacterial membrane bioenergetics. Among these systems, proton-motive force (PMF) dependent Resistance, Nodulation–Division (RND) efflux pumps, such as AcrAB-TolC, play a central role in both intrinsic and acquired antibiotic resistance by expelling structurally diverse antimicrobial agents. Recent evidence indicates that efflux pumps are not merely drug extrusion devices but also key regulators of bacterial physiology, influencing membrane potential, redox balance, metabolic state, stress adaptation, and growth-phase transitions. Structural and mechanistic advances have uncovered conserved allosteric sites within RND pumps that are distinct from substrate-binding pockets, enabling the development of allosteric efflux pump inhibitors (EPIs) that disrupt conformational cycling and proton relay without competing with antibiotics. Pyridylpiperazine-based inhibitors, including BDM-series compounds, have demonstrated potent efflux inhibition, antibiotic potentiation, and *in-vivo* efficacy in preclinical models. This review integrates current knowledge on efflux pump architecture, PMF-driven transport mechanisms, membrane potential dynamics, and allosteric inhibition, emphasizing the therapeutic potential of dual-targeting strategies that combine efflux inhibition with bioenergetics disruption. This study also focused on translational challenges, including drug penetration, resistance evolution, and pharmacokinetic constraints, and future directions for incorporating efflux–bioenergetic targeting into next-generation antimicrobial discovery pipelines to restore antibiotic efficacy against MDR Gram-negative pathogens.

## 1 Introduction

The escalating prevalence of multi-drug resistance (MDR) Gram-negative bacterial pathogens constitutes one of the most critical global health challenges of the 21st century. Clinically significant species, including *Pseudomonas aeruginosa*, *Acinetobacter baumannii*, and carbapenem-resistant *Enterobacterales*, have exhibited a remarkable capacity for the rapid acquisition and horizontal dissemination of antimicrobial resistance determinants, resulting in severely limited therapeutic options and substantially increased morbidity and mortality worldwide (Macesic et al., 2025). The intrinsic resilience of Gram-negative bacteria is largely attributable to their unique cell envelope architecture, particularly the outer membrane, which acts as a formidable permeability barrier to many antibiotics. This intrinsic resistance is further compounded by multiple acquired mechanisms, including target modification, enzymatic inactivation of antibiotics, and the overexpression of multidrug efflux systems (Gauba and Rahman, 2023). Reflecting this growing threat, the World Health Organization (WHO) has designated several Gram-negative pathogens as priority organisms due to their escalating resistance to last-resort antibiotics such as carbapenems and polymyxins (Gauba and Rahman, 2023). Efforts to combat MDR Gram-negative infections are severely constrained by the limited pipeline of novel antibiotics and the rapid evolution of resistance even against newly introduced agents. These limitations underscore the urgent need for alternative and adjunctive therapeutic strategies, including phage therapy, anti-virulence approaches, and rational antibiotic combination therapies (Mascellino et al., 2024; Macesic et al., 2025). In parallel, robust infection control measures and antimicrobial stewardship programs remain essential to curtail the dissemination of resistant strains in both healthcare and community settings (Kavya et al., 2024). Among the diverse resistance mechanisms employed by Gram-negative bacteria, multidrug efflux pumps play a central and multifaceted role. These membrane-embedded transport systems actively expel a wide spectrum of structurally unrelated antimicrobial agents, thereby reducing intracellular drug concentrations to sub-inhibitory levels. Nodulation–Division (RND) family efflux pumps, such as AcrAB–TolC in *Escherichia coli* and *Salmonella* spp., which are powered by the proton motive force (PMF) across the inner membrane, are tripartite complexes that span the Gram-negative cell envelope to actively expel toxic compounds including antibiotics (Gaurav et al., 2023; Kumawat et al., 2023). The functional dependence of efflux activity on membrane energetics establishes a critical link between antimicrobial resistance and bacterial energy metabolism. Recent transcriptomic, biophysical, and physiological studies have revealed that perturbation or inhibition of major RND efflux pumps, including AcrB, results in altered proton flux across the inner membrane, leading to membrane hyperpolarization (Whittle et al., 2024). This energetic imbalance influences global regulatory networks, such as the ArcBA two-component system, thereby reshaping bacterial redox homeostasis, delaying entry into stationary phase, and modulating stress adaptation. Stationary-phase cells typically exhibit reduced antibiotic permeability and enhanced tolerance, these findings highlight an underappreciated role of efflux pumps in governing bacterial physiological states beyond drug extrusion (Whittle et al., 2024). Moreover, efflux inhibition–induced alterations in membrane potential may amplify antibiotic-mediated reactive oxygen species (ROS) production, further potentiating bactericidal activity. These observations position efflux pumps as integral hubs linking antimicrobial resistance, membrane energetics, metabolic regulation, and stress responses. Their dual function as both transporters and modulators of cellular bioenergetics suggests that targeting efflux systems could dismantle resistance at both physical and energetic levels.

Accordingly, the development of efflux pump inhibitors that simultaneously disrupt drug export and proton motive force utilization represents a promising strategy for restoring antibiotic efficacy against MDR Gram-negative pathogens (Gaurav et al., 2023; Kumawat et al., 2023). The objective of this review is to provide a comprehensive and integrative analysis of the roles of multi-drug efflux pumps and membrane energetics in shaping antimicrobial resistance in Gram-negative bacteria. Moving beyond conventional efflux-centered paradigms, this review integrates recent insights into efflux-mediated regulation of metabolism, stress responses, virulence, and resistance evolution. Furthermore, it critically evaluates current progress and challenges in efflux pump inhibitor development, emphasizing the necessity of targeting both efflux machinery and its energetic drivers (Thakur et al., 2021). This review provides a unified framework that redefines efflux pumps as central regulators of bacterial adaptation rather than passive determinants of multi-drug resistance.

## 2 Major efflux pump families in bacteria

**FIGURE 1:**
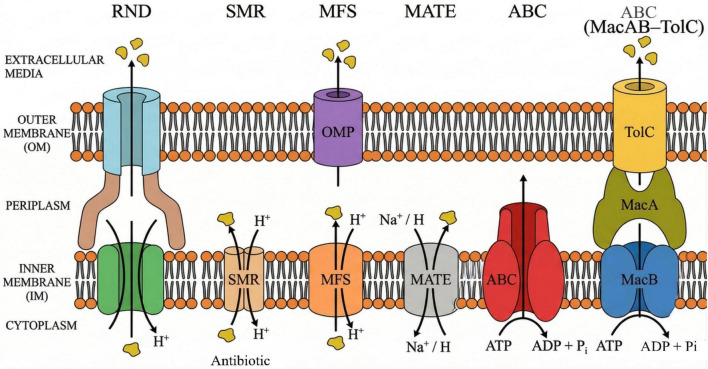
Schematic overview of the major bacterial efflux pump families. This figure provides a schematic representation of the structural arrangement and transport mechanism of bacterial RND, SMR, MFS, MATE, and ABC transporters. In RND transporters, a tripartite structure spanning both membranes uses the proton motive force. SMR and MFS transporters use a proton gradient, whereas MATE transporters use a Na^+^ or H^+^ gradient. ABC transporters use ATP hydrolysis. The MacAB-TolC system is an ABC-type tripartite efflux pump consisting of the inner membrane protein MacB, the periplasmic protein MacA, and the outer membrane protein TolC. IM, inner membrane; OM, outer membrane; OMP, outer membrane protein.

Bacterial efflux pumps, critical contributors to multidrug resistance, are classified mainly into five major families based on their energy sources and structural features such as resistance-nodulation-cell division (RND), major facilitator superfamily (MFS), ATP-binding cassette (ABC) transporters, multidrug and toxic compound extrusion (MATE), and small multidrug resistance (SMR) families (Chetri, 2023; Gaurav et al., 2023). The RND family, primarily found in Gram-negative bacteria, includes tripartite assemblies such as AcrAB-TolC, which expel a wide range of antibiotics and toxic compounds using the proton motive force for energy (Chetri, 2023; Duffey et al., 2024). The MFS pumps are ubiquitous across bacterial species, functioning primarily as single-component transporters that harness proton gradients and are responsible for exporting structurally diverse antibiotic substrates, such as tetracyclines and chloramphenicol (Chetri, 2023; Gaurav et al., 2023). In contrast to other efflux pumps, ABC transporters are mechanistically distinct in their use of ATP hydrolysis to energize substrate transport. A prominent efflux system in Gram-negative bacteria such as MacAB-TolC system, which is known to act as an ABC-type tripartite efflux system, where an inner membrane ATP-binding cassette system, MacB, is assisted by a periplasmic adaptor protein, MacA, and an outer membrane channel (TolC), to export macrolide compounds and other substrates (Chetri, 2023; Duffey et al., 2024). The MATE family utilizes sodium or proton antiport to extrude cationic drugs, including fluoroquinolones and aminoglycosides, playing a crucial role in resistance in multiple bacterial genera (Chetri, 2023; Gaurav et al., 2023). Finally, SMR pumps are typically small homodimers with narrow substrate recognition profiles, known for expelling antiseptics and disinfectants, contributing to resistance against quaternary ammonium compounds and some antibiotics (Chetri, 2023). Collectively, these families illustrate diverse structural and functional adaptations that enable bacterial survival under antimicrobial pressure. Understanding these distinct pump classes provides a framework for designing targeted inhibitors to mitigate efflux-mediated resistance, an essential strategy for restoring the clinical efficacy of existing antibiotics (Chetri, 2023; Duffey et al., 2024). Different efflux pumps with their other characteristics are given in the below Figure 1 and Table 1.

**TABLE 1:** Different efflux pumps and their characteristics.

Efflux pump family	Energy source	Structural features	Substrate specificity	Representative pumps	References
RND	Proton Motive Force	Tripartite complex spanning the inner membrane to the outer membrane (transporter, adaptor, channel)	Broad, including β-lactams, quinolones, macrolides, and tetracyclines	AcrAB-TolC (*E. coli*), MexAB-OprM (*P. aeruginosa*)	(Jang, 2023; Gurvic and Zachariae, 2024)
MFS	Proton Gradient	Single-component with 12–14 transmembrane helices	Moderate, diverse antibiotics, including tetracycline, chloramphenicol	EmrD, MdfA	(Huang et al., 2022)
SMR	Proton Gradient	Small, four transmembrane helices, often dimers	Limited, small cationic drugs, antiseptics	EmrE	(Duffey et al., 2024)
ABC	ATP Hydrolysis	Two-component (transmembrane + nucleotide-binding domains)	Diverse, including peptides, lipids, and antibiotics	MacAB-TolC (some species)	(Duffey et al., 2024)
MATE	Sodium or Proton Gradient	12 transmembrane helices	Fluoroquinolone, aminoglycosides	NorM (V. cholerae), DinF-like pumps	(Gurvic and Zachariae, 2024)

## 3 Structural and functional features of clinically relevant pumps

**FIGURE 2:**
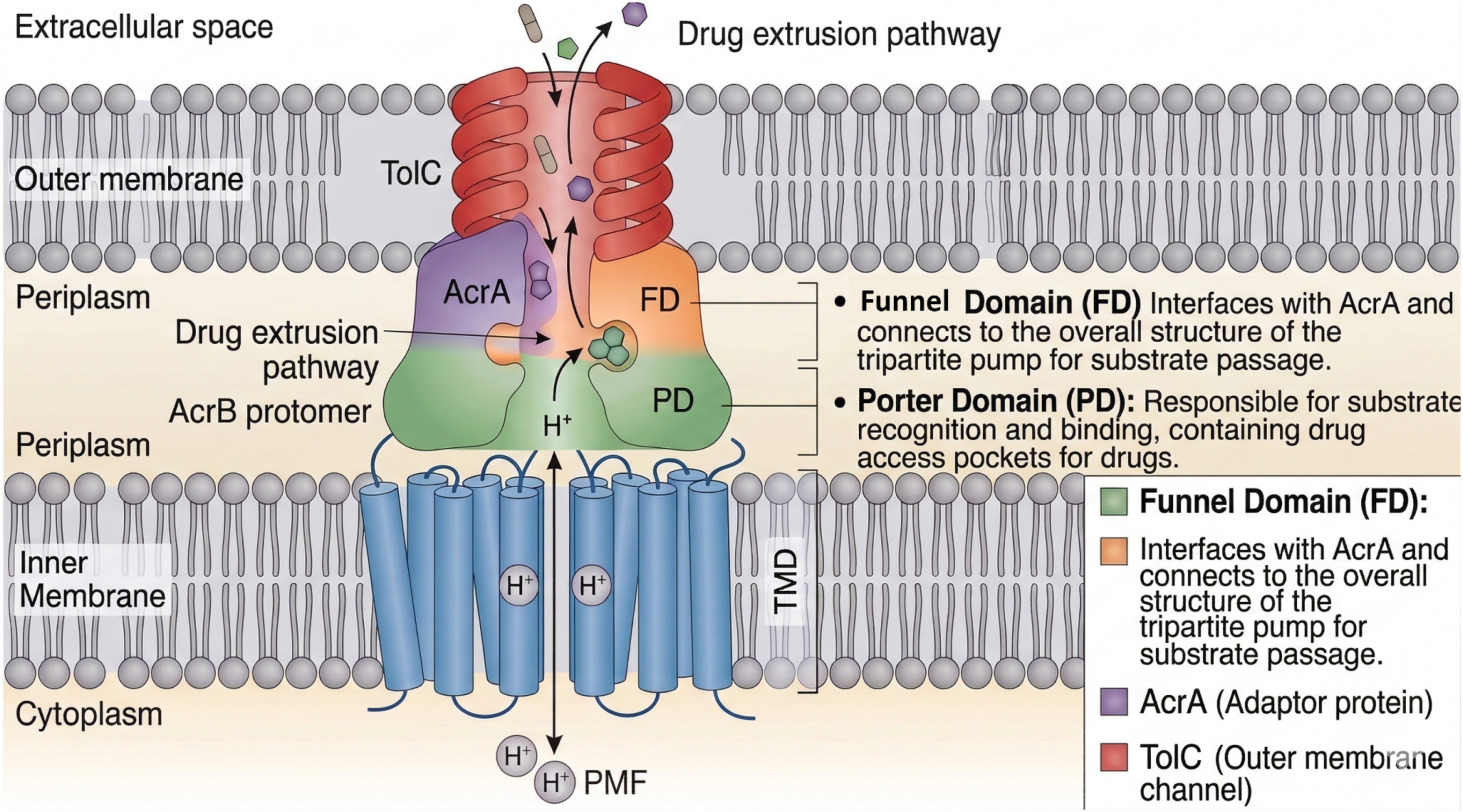
Schematic representation of the tripartite AcrAB–TolC efflux system spanning the inner membrane, periplasm, and outer membrane of Gram-negative bacteria. AcrB utilizes the proton motive force (PMF) through its transmembrane domain (TMD), while the porter domain (PD) and funnel domain (FD) mediate substrate binding and connection to AcrA. AcrA links AcrB to the outer membrane channel TolC, enabling drug extrusion to the extracellular space.

The AcrAB-TolC efflux pump is a prototypical and extensively studied tripartite resistance-nodulation-division (RND) family multidrug efflux system predominantly found in Gram-negative bacteria such as *Escherichia coli* and *Salmonella* sp. This pump significantly contributes to multidrug resistance by extruding a broad spectrum of antibiotics and toxic compounds, thereby decreasing intracellular drug accumulation and efficacy (Vargiu et al., 2022; Sharma et al., 2024). Structurally, AcrAB-TolC comprises three components: AcrB, an inner membrane RND transporter that forms a homotrimer; AcrA, a periplasmic membrane fusion protein (MFP) that bridges AcrB and TolC; and TolC, an outer membrane channel forming the exit duct (Vargiu et al., 2022; Sharma et al., 2024). Each AcrB protomer consists of three domains—the transmembrane domain (TMD), which harnesses the proton motive force to energize transport, the porter domain (PD) responsible for substrate recognition and binding, and the funnel domain (FD) that interfaces with AcrA (Vargiu et al., 2022). The pump operates through a functional rotation mechanism that cycles the protomers through distinct conformational states, known as access (loose), binding (tight), and extrusion (open), enabling substrate (antibiotic) capture, transport, and expulsion, respectively (Nishino et al., 2021; Vargiu et al., 2022). TolC forms a long α/β-barrel channel that undergoes conformational changes from closed to open states to release substrates into the extracellular space, coordinated by conformational shifts in AcrA and AcrB during the efflux cycle (Nishino et al., 2021; Sharma et al., 2024). This tripartite assembly enables the efflux of structurally diverse molecules, including antibiotics from multiple classes, detergents, dyes, and bile salts, contributing to broad substrate specificity (Vargiu et al., 2022; Ren et al., 2024). The precise interaction of the AcrA hexamer with AcrB and TolC forms a continuous conduit spanning both membranes, preventing leakage and enhancing specificity (Vargiu et al., 2022). Recent structural studies, employing cryo-electron microscopy and X-ray crystallography, have elucidated the atomic-level details of the pump’s assembly and operational dynamics, laying the groundwork for the rational design of efflux pump inhibitors targeting key conformational states and substrate binding sites (Figure 2; Nishino et al., 2021; Kavanaugh et al., 2024).

## 4 Contribution of efflux pumps to intrinsic and acquired resistance

Efflux pumps represent a fundamental mechanism of intrinsic and acquired resistance to a broad spectrum of antimicrobial agents. Intrinsic resistance arises from the basal or constitutive expression of efflux systems that actively extrude antibiotics and toxic compounds, thus lowering intracellular drug concentrations to sub-lethal levels (Soto, 2013; Sharma et al., 2024). For instance, the RND-family efflux pumps, such as AcrAB-TolC in *Escherichia coli* and Mex systems in *Pseudomonas aeruginosa*, contribute significantly to this innate defense by transporting diverse substrates, including β-lactams, fluoroquinolones, and tetracyclines (Dulanto Chiang and Dekker, 2024; Sharma et al., 2024). Acquired resistance mechanisms often involve the upregulation or mutation-driven overexpression of efflux pumps, which enhances substrate expulsion and enables bacteria to survive higher concentrations of antibiotics (Chetri, 2023; Li et al., 2025). Additionally, efflux pumps play a key role in the rising of resistance against the antibiotics within the bacterial cells. Such sub inhibitory exposure to antibiotics may promote accumulation of secondary mutations in the antibiotics targets including mutations in DNA gyrase or topoisomerase IV which confer resistance to fluoroquinolones and changes in the ribosomal proteins linked to macrolide resistance or changes in the penicillin binding proteins that prevent beta lactam binding. This process finally leads to the development of target mediated antibiotic resistance (Soto, 2013; Chetri, 2023). Beyond direct drug extrusion, efflux pumps also play roles in biofilm formation and the secretion of virulence factors, indirectly promoting resistance by creating protective microbial communities that are less susceptible to antibiotics (Kumawat et al., 2023; Sharma et al., 2024). Efflux pumps proteins in the bacteria influence biofilm formation by expelling some QS molecules such as AHLs (acyl-homoserine lactones) and other signaling molecules which are considered as a virulence factors and those molecules also regulate concerned gene regulation involved in the biofilms maturation and EPS production which are key constituents of biofilm (Soto, 2013). These biofilms in the bacteria increase the tolerance to antibiotics by reducing the entry of antibiotics in the bacterial cell. Efflux pumps proteins also reported for the secretion of virulence factors, toxins, adhesin proteins and regulate global network of bacteria. These all activities of the bacteria contribute to the formation of microbial communities which help to exist in the presence of heavy attacks of antimicrobial compounds by enhancing collective resistance to different stresses and antimicrobial molecules (Du et al., 2018; Sharma et al., 2019). The dual role of these pumps in both intrinsic protection and the acquisition of enhanced resistance underscores the critical need for novel therapeutic strategies targeting efflux systems to restore antibiotic efficacy (Chetri, 2023; Dulanto Chiang and Dekker, 2024).

## 5 Why classical efflux pump inhibitors failed in clinical translation?

Early efforts to combat multidrug resistance through classical efflux pump inhibitors (EPIs) were largely based on competitive inhibition of substrate-binding sites within the multidrug transporters. Despite promising *in-vitro* potentiation of antibiotics, most classical EPIs failed to progress into clinical use due to a combination of pharmacological, mechanistic, and toxicological limitations. One major obstacle was substrate competition, as competitive EPIs must directly contend with high intracellular or periplasmic concentrations of antibiotics and endogenous metabolites. In Gram-negative bacteria, the remarkable substrate polyspecificity and high turnover rate of RND pumps such as AcrAB-TolC render competitive inhibition inherently inefficient, particularly under clinical drug exposure conditions where substrate overload can easily outcompete inhibitors (Opperman and Nguyen, 2015; Rouvier et al., 2025). Another critical limitation was poor permeability across the Gram-negative outer membrane. Many early EPIs, including phenylalanine-arginine β-naphthylamide (PAβN), exhibited limited penetration and were often themselves substrates of efflux pumps, resulting in insufficient intracellular accumulation at target sites (Duffey et al., 2024; Rouvier et al., 2025). Furthermore, classical EPIs frequently displayed dose-limiting toxicity, arising from off-target interactions with mammalian membranes, ion channels, or transporters, thereby narrowing their therapeutic window and precluding systemic administration (Thakur et al., 2021; Duffey et al., 2024). These toxic effects were particularly pronounced for membrane-active or amphipathic compounds, which required reaching effective inhibitory concentrations. From a resistance standpoint, classical EPIs were also vulnerable to rapid resistance development, as mutations in substrate-binding pockets or pump overexpression readily diminished inhibitor efficacy without substantially compromising pump function (Opperman and Nguyen, 2015; Thomson and Saadhali, 2025). Additionally, competitive EPIs typically failed to disrupt the energetic coupling between efflux activity and the proton motive force (PMF), allowing bacteria to maintain efflux competence through metabolic adaptation or compensatory upregulation of alternative transporters (Nishino et al., 2021; Rouvier et al., 2025). Collectively, these shortcomings highlighted the need for mechanistically distinct strategies—such as allosteric inhibition—that bypass substrate competition, exploit conserved conformational control points, and destabilize efflux–bioenergetic coupling more effectively in Gram-negative pathogens.

## 6 Advantages of allosteric inhibition over competitive inhibition

**FIGURE 3:**
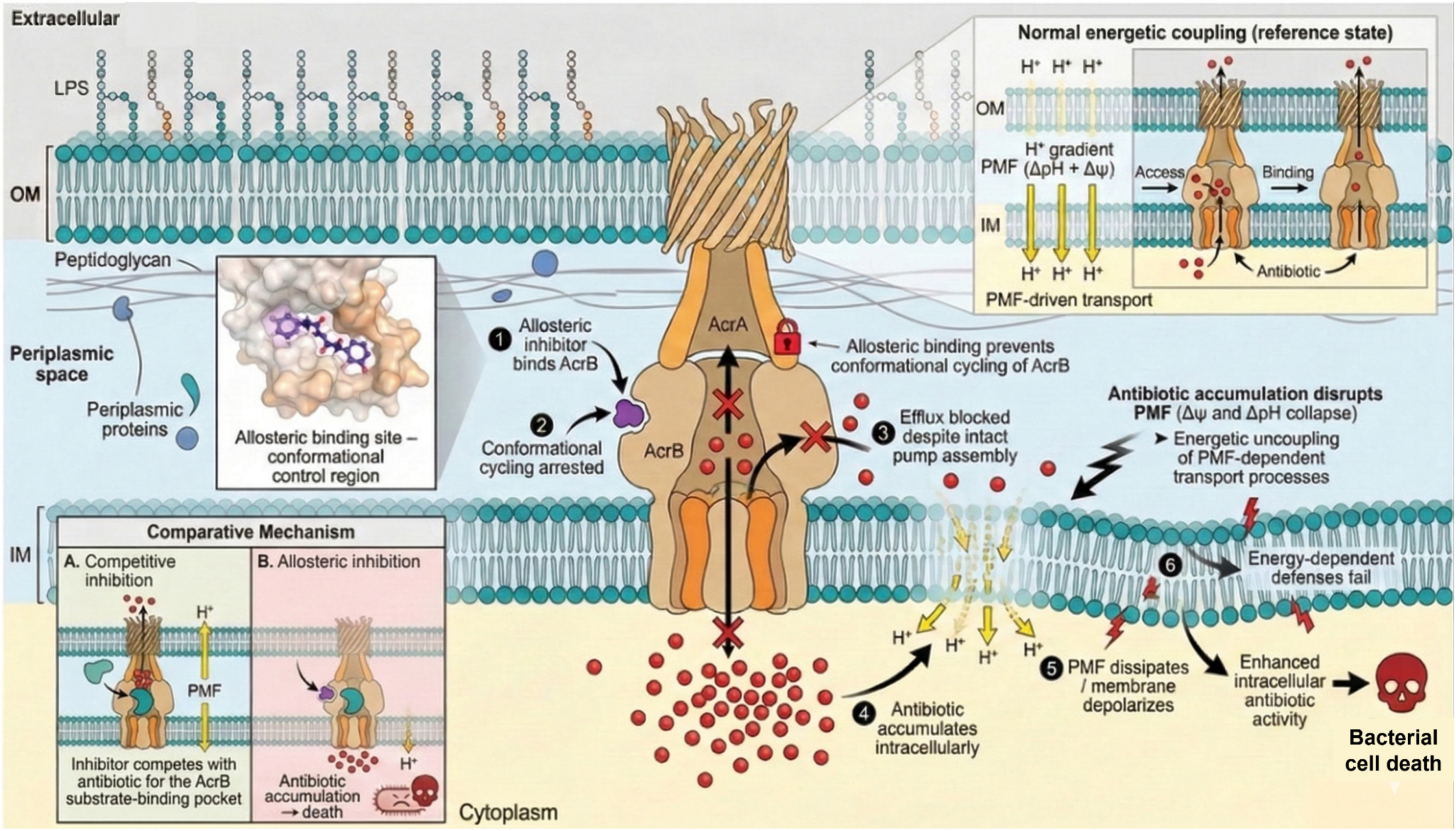
An allosteric inhibitor binds AcrB and locks its conformational cycling, blocking antibiotic efflux through the AcrAB—TolC pump. As antibiotics accumulate inside the cell, they collapse the proton-motive force (ΔΨ and ΔpH) and depolarize the membrane. Loss of PMF further disables energy-dependent defenses, amplifying antibiotic activity and leading to bacterial cell death.

Allosteric inhibition of bacterial efflux pumps is characterized by the binding of an inhibitor molecule to a site distinct from the active or substrate-binding site on the pump protein, known as the allosteric site. This binding induces conformational changes in the pump structure, disrupting its functional cycle and thereby reducing or abolishing its ability to expel antimicrobial agents from the bacterial cell (Plé et al., 2022). Unlike competitive inhibitors, which directly compete for occupancy at the active site, allosteric inhibitors modulate pump activity indirectly by altering the topology or dynamics of the efflux machinery (Opperman and Nguyen, 2015; Rouvier et al., 2025). This mode of inhibition often leads to a non-competitive or uncompetitive phenotype whereby inhibition efficiency is independent of substrate concentration, providing a consistent suppression of efflux activity. One major advantage of allosteric inhibition over competitive inhibition is its potential to overcome the intrinsic limitation of substrate competition. Furthermore, allosteric inhibitors can target conserved allosteric pockets critical for the efflux pump’s conformational transitions during the efflux cycle, thereby potentially providing a broader spectrum of inhibition against multiple pump substrates (Plé et al., 2022; Rouvier et al., 2025). Additionally, the unique binding to allosteric sites reduces the chance of cross-resistance with substrates that share the active site, offering new avenues for circumventing multidrug resistance in Gram-negative bacteria. Recent structural insights into Unlike competitive inhibitor RND family efflux pumps, such as AcrAB-TolC, have revealed novel allosteric pockets in the transmembrane domain and periplasmic regions, which, when occupied by selective inhibitors, prevent essential conformational cycling necessary for substrate extrusion (Figure 3; Plé et al., 2022). Antibiotic accumulation inside the bacterial cells can disrupt the Proton Motive Force (PMF) by increasing proton concentration inside the cell. After the accumulation of antibiotics, protons started to accumulate in the inner membrane of the bacteria, leads to directly damage the cytoplasmic membrane or indirectly affect components of the Electron Transport Chain, which lead to dissipation of the transmembrane proton gradient (ΔpH) and membrane potential (Δψ). Further transmembrane proton gradient (ΔpH) and membrane potential (Δψ) collapse and energy dependent defenses fail which lead to bacterial death (Plé et al., 2022). Since PMF is essential for ATP synthesis, nutrient transport, and the activity of energy-dependent systems such as RND efflux pumps, excessive intracellular accumulation of antibiotics ultimately collapses the electrochemical gradient and compromises cellular viability (Chetri, 2023).

## 7 Recent advances in the identification of allosteric binding sites on efflux pumps

Recent advances in identifying allosteric binding sites on bacterial efflux pumps have significantly enhanced the understanding of their inhibition mechanisms, particularly within the resistance-nodulation-division (RND) family of efflux pumps, such as AcrB. High-resolution cryo-electron microscopy (cryo-EM) and X-ray crystallography studies have unveiled novel allosteric sites distinct from the canonical substrate-binding pockets, predominantly located in the transmembrane domain, porter domain, and at subunit interfaces of the tripartite efflux machinery (Wang et al., 2017; Plé et al., 2022). These structural insights have established that binding of allosteric inhibitors at these sites modulates the conformational dynamics essential for pump function, disrupting proton motive force coupling and substrate transport without directly competing with antibiotics at the active site (Plé et al., 2022; Roy et al., 2024). Plé et al., identified a unique allosteric binding pocket within the transmembrane region of AcrB targeted by pyridylpiperazine-based inhibitors (Plé et al., 2022). This binding site includes residues critical for proton relay, and binding to this pocket effectively halts the efflux pump’s catalytic cycle, demonstrating a potent mechanism of inhibition that bypasses the limitations of competitive inhibition (Plé et al., 2022). Complementing these structural discoveries, molecular dynamics simulations and computational network analyses have mapped allosteric communication pathways linking the transmembrane proton-translocation domain with the periplasmic substrate-binding porter domain.

## 8 Chemical classes of allosteric efflux pump inhibitors

Classical efflux pump inhibitors (EPIs) are compounds that inhibit bacterial multidrug efflux pumps. Classical inhibitors generally act by blocking the substrate-binding site on efflux pumps and interfering with pump assembly and function (Sharma et al., 2019). The combined use of classical efflux pump inhibitors (EPIs) with pump substrates is under exploration to overcome efflux-mediated multidrug resistance. Phenylalanine-arginine β-naphthylamide (PAβN) is a well-studied classical EPI that is routinely combined with fluoroquinolone antibiotics, but few studies have assessed its utility in combination with β-lactam antibiotics (Compagne et al., 2023). PAβN inhibits the three RND pumps of *P. aeruginosa* (MexAB-OprM, MexCD-OprJ, and MexEF-OprN) and is also a substrate of these pumps, indicating it acts as a competitive inhibitor. This classical inhibitor, β-naphthylamide (PAβN), binds competitively and prevents drug extrusion. Reserpine is also a classical efflux pump inhibitor, which also blocks substrate binding sites of efflux pumps (Shaheen et al., 2019).

Several chemical classes of allosteric efflux pump inhibitors (EPIs) have emerged as promising agents to combat multidrug resistance mediated by RND-type efflux pumps, particularly AcrAB-TolC in Gram-negative bacteria. Among these, the pyridylpiperazines represent a pioneering family that binds to a novel allosteric site in the transmembrane domain of the AcrB L protomer, distinct from the classical drug-binding pockets (Table 2).

**TABLE 2:** Allosteric efflux pumps inhibitors.

Inhibitor class	Example compound	Target efflux pump	Potency (IC50/EC50)	Spectrum of activity	Mode of action	Clinical translation prospects	Structure	References
Pyridylpiperazines	MBX2319	AcrB	0.5–2 μM	Broad (Gram-negative)	Binds hydrophobic trap, disrupts conformational cycle, prevents substrate extrusion	Preclinical, promising	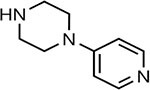	(Opperman et al., 2014)
D13-9001	D13-9001	MexB	0.1–1 μM	*Pseudomonas aeruginosa*	Binds novel allosteric site, induces conformational changes, blocks efflux pathway	Preclinical, advanced	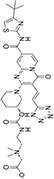	(Nakashima et al., 2013)
BDM88855	BDM88855	AcrB	0.2–1 μM	Broad (Gram-negative)	High-affinity binding to the allosteric site stabilizes the inactive conformation, inhibits pump function	Preclinical, promising	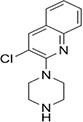	(Blair et al., 2015)

These inhibitors, exemplified by compounds like BDM73185 and its optimized analog BDM88855, act by sterically preventing crucial conformational changes in the pump’s catalytic cycle, thereby disabling the proton relay essential for drug efflux (Plé et al., 2022; Vieira Da Cruz et al., 2024). BDM88855, a lead compound in the BDM series, exhibits potent activity by directly binding the transmembrane allosteric pocket, stabilizing an inactive pump conformation, and significantly boosting antibiotic potency against AcrAB-TolC substrates such as chloramphenicol, tetracycline, and ciprofloxacin (Plé et al., 2022; Jiménez-Castellanos et al., 2023). The protonated piperazine moiety of BDM88855 forms critical interactions with catalytic residues within the pump, blocking the proton motive force essential for transport and disrupting the functional rotation mechanism (Table 2; Plé et al., 2022). Besides pyridylpiperazines, other allosteric EPIs have been identified, including arylpiperazines like NMP (1-(1-naphthylmethyl)-piperazine) and members of the MBX series, which target hydrophobic or transmembrane pockets proximal to drug-binding sites, thereby halting substrate extrusion through allosteric inhibition (Jang, 2023; Duffey et al., 2024). These inhibitors offer advantages over competitive EPIs by binding to less conserved allosteric sites, thereby reducing the development of resistance and allowing for the simultaneous inhibition of multiple efflux pump conformations (Plé et al., 2022; Jiménez-Castellanos et al., 2023).

## 9 Mechanisms of action of efflux pumps inhibitors at the molecular and structural levels

Efflux pumps transport proteins of bacteria which participate in extrusion of substrates (generally antibiotics) from the cellular interior to the external environment (Sharma et al., 2019). The proton gradient or the ATPase that supplies energy to these pumps has been tried as targets of various EPIs (Sharma et al., 2019). Carbonyl cyanide-m-chlorophenylhydrazone (CCCP) is an EPI ionophore that disrupts the proton motive force (PMF) by affecting both its components, Δψ and ΔpH (Bhattacharyya et al., 2017). Verapamil is a small molecule that acts as an ion channel blocker and is used in the treatment of hypertension. Studies in *Mycobacterium tuberculosis* have shown that verapamil potentiates the activity of bedaquiline and ofloxacin (Singh et al., 2011; Gupta et al., 2014).

Allosteric efflux pump inhibitors (EPIs) exert their effects by binding to regulatory sites distinct from the substrate-binding pockets, inducing conformational changes that disrupt the functional cycle of multidrug efflux pumps, particularly those of the RND family, such as AcrAB-TolC (Sjuts et al., 2016; Plé et al., 2022). At the molecular level, many EPIs target the transmembrane domain (TMD; made of 12 transmembrane helices) of the inner membrane transporter AcrB, especially the L protomer’s allosteric pocket, where critical proton relay residues reside (Plé et al., 2022). The binding of inhibitors, such as pyridylpiperazines or BDM88855 changes the conformation of pumps in non-functional form that prevents the necessary conformational transitions between the access (L), binding (T), and extrusion (O) states for substrate transport (Sjuts et al., 2016; Plé et al., 2022; Jiménez-Castellanos et al., 2023). This blockage abrogates the Proton Motive Force (PMF)-driven functional rotation that effectively “freezing” the pump and halting drug extrusion (Wang et al., 2017; Plé et al., 2022). Molecular dynamics simulations also focus on inhibitors-based restriction of transmembrane helices flexibility, preventing the remodeling of the drug-binding pocket necessary for substrate access and export (Sjuts et al., 2016; Jamshidi et al., 2018).

## 10 Mutagenesis and crystallography studies supporting allosteric inhibition

Evidence from site-directed mutagenesis and high-resolution crystallography has been pivotal in substantiating the mechanistic basis of allosteric inhibition of multidrug efflux pumps, particularly AcrAB-TolC and related RND transporters. Structural analyses employing co-crystal structures of pyridylpiperazine-based inhibitors such as BDM88855 bound to AcrB reveal a unique allosteric pocket located within the transmembrane domain of the L protomer, spatially distinct from classical substrate-binding sites (Plé et al., 2022; Jiménez-Castellanos et al., 2023). This crystallographic insight demonstrated specific interactions between the inhibitor and critical proton-relaying residues, including aspartates essential for harnessing the proton motive force, thereby impeding the energy transduction necessary for pump operation (Plé et al., 2022).

Mutagenesis studies have further corroborated the allosteric mechanism by identifying pump variants with point mutations in transmembrane helices that abolish inhibitor binding and confer resistance to allosteric EPIs (Plé et al., 2022; Jiménez-Castellanos et al., 2023). For example, substitutions such as S450P and A446P in TM5 of AcrB were discovered in resistant *Escherichia coli* mutants exposed to pyridylpiperazine inhibitors, highlighting the functional importance of these residues in the allosteric binding pocket for inhibitor efficacy (Plé et al., 2022). These mutations, located distant from the substrate-binding pockets, support a mechanism in which allosteric inhibitors disrupt conformational cycling through steric hindrance. Complementary mutagenesis mapping, combined with thermodynamic stability assays, demonstrated that allosteric inhibitors stabilize pump conformations unfavorable for substrate transport. This stabilization was manifested as increased thermal shift values for wild-type complexes but not for resistant mutants, confirming direct inhibitor engagement at the allosteric site (Plé et al., 2022; Jiménez-Castellanos et al., 2023).

## 11 Allosteric EPIs and their potency, spectrum, and *in-vivo* efficacy

Leading allosteric efflux pump inhibitors (EPIs), particularly those based on the pyridylpiperazine (PyrPip) scaffold, such as BDM88855 and its analogs, exhibit potent inhibition of RND-type pumps like AcrAB-TolC, with broad-spectrum activity against clinically relevant Gram-negative pathogens, including *Escherichia coli*, *Klebsiella pneumoniae*, and *Acinetobacter baumannii* (Plé et al., 2022; Jiménez-Castellanos et al., 2023; Vieira Da Cruz et al., 2024). These PyrPip EPIs increase the intracellular accumulation of a wide range of antibiotics, leading to 2–8-fold enhancement in antibiotic efficacy *in-vitro*, particularly with substrates such as chloramphenicol, tetracycline, erythromycin, and ciprofloxacin (Plé et al., 2022). BDM88855, a lead compound, exhibits micromolar potency with half-maximal effective concentration (EC50) values in the low micromolar range (∼1–5 μM) across multiple efflux pump assays (Plé et al., 2022; Jiménez-Castellanos et al., 2023). *In-vitro* studies elucidate its selectivity for RND efflux pumps without affecting non-substrate antibiotics, underscoring its specificity (Plé et al., 2022). The spectrum of these inhibitors encompasses multiple multidrug-resistant strains, making them promising candidates for restoring antibiotic susceptibility where efflux-mediated resistance predominates (Jiménez-Castellanos et al., 2023; Vieira Da Cruz et al., 2024). Critically, proof-of-concept *in-vivo* studies validate the therapeutic potential of PyrPip EPIs. For example, BDM91288, an optimized analog of BDM88855, significantly potentiated the efficacy of levofloxacin in a murine model of *K. pneumoniae* pulmonary infection, reducing bacterial load and increasing survival rates compared to antibiotic treatment alone (Plé et al., 2022; Vieira Da Cruz et al., 2024). Pharmacokinetic analyses highlight favorable metabolic stability and target tissue distribution, although further optimization is underway to enhance organ penetration and plasma retention (Vieira Da Cruz et al., 2024). Other allosteric EPIs, such as the MBX series, also show promising spectrum and *in-vivo* efficacy, with compounds like MBX3796 exhibiting improved ADME properties and activity against resistant *Pseudomonas aeruginosa* strains (Duffey et al., 2024). Collectively, these data underscore the progress toward clinically viable allosteric efflux pump inhibitors that can combat multidrug resistance in Gram-negative pathogens.

## 12 Role of membrane potential in efflux pump function and drug resistance

### 12.1 Overview of bacterial membrane potential and proton motive force

Efflux pumps of the RND family, among others, harness the energy from PMF to actively extrude a broad spectrum of antibiotics and harmful metabolites out of bacterial cells, thereby contributing critically to intrinsic and acquired antimicrobial resistance (Wan et al., 2023; Yang et al., 2023). The magnitude and maintenance of the membrane potential and proton gradient directly influence efflux efficiency, impacting intracellular drug accumulation and bacterial susceptibility (Wan et al., 2023). Recent investigations show bacteria dynamically regulate PMF under stress conditions, such as nutrient starvation or antibiotic exposure, to sustain efflux pump function, thus facilitating phenotypic tolerance and survival (Wan et al., 2023; Biquet-Bisquert et al., 2024). Moreover, the membrane potential itself contributes to the electrochemical driving force needed for proton translocation in pumps like AcrB, which couples proton influx to substrate expulsion, underpinning the proton relay mechanism critical for efflux pump catalytic cycles (Wan et al., 2023; Yang et al., 2023). Perturbation of PMF, for example, by protonophores or environmental stresses, leads to compromised efflux pump activity, higher intracellular antibiotic concentrations, and increased bacterial susceptibility, highlighting the essential role of membrane energetics in antimicrobial resistance (Yang et al., 2023; Biquet-Bisquert et al., 2024). Collectively, this bioenergetic foundation underscores the importance of targeting membrane potential and PMF in strategies aiming to overcome efflux-mediated drug resistance in bacterial pathogens (Yang et al., 2023).

### 12.2 Energy coupling between PMF and efflux pump catalytic cycles

The catalytic cycles of multidrug efflux pumps, especially those in the RND and MFS families, are tightly coupled to the proton motive force (PMF) across the bacterial cytoplasmic membrane (Chetri, 2023; Li et al., 2024). This energy is transduced by dedicated proton relay networks within the inner membrane transporter (e.g., AcrB for RND pumps), where conserved charged residues (such as D407, D408, K940 in AcrB) cycle through protonation and deprotonation events to propel substrate extrusion and drive conformational transitions within the pump protomers (Plé et al., 2022; Li et al., 2024). Recent cryo-EM and mutagenesis studies highlight that substrate binding and release are coordinated with the passage of protons through the transmembrane domain, linking drug export directly to PMF-driven conformational cycling (Plé et al., 2022; Li et al., 2024). For example, each AcrB protomer transitions through loose (L), tight (T), and open (O) states, each of which is associated with protonation steps and accompanied by substrate recognition, binding, and expulsion, thereby ensure the vectorial transport of antibiotics out of the cell (Plé et al., 2022). Blockage or dissipation of the PMF—whether by membrane-acting antibiotics, metabolic inhibitors, or targeted small-molecule EPIs—interrupts proton translocation, “freezing” the pump in inactive conformations and leading to elevated intracellular drug concentrations (Gerry et al., 2025). Critically, membrane potential and ΔpH do not always act as a single unit; recent models show that they can individually fine-tune the overall pump throughput and substrate specificity, underlining the non-equilibrium and adaptable nature of energy transduction in efflux-mediated resistance (Li et al., 2024; Gerry et al., 2025). Importantly, these mechanistic insights reveal that PMF perturbation can selectively reduce efflux efficiency and sensitize bacteria to antibiotics, highlighting PMF-coupled transport as a compelling target for adjuvant therapeutic strategies (Chetri, 2023).

### 12.3 Experimental and computational studies linking membrane potential dynamics to efflux modulation

**FIGURE 4:**
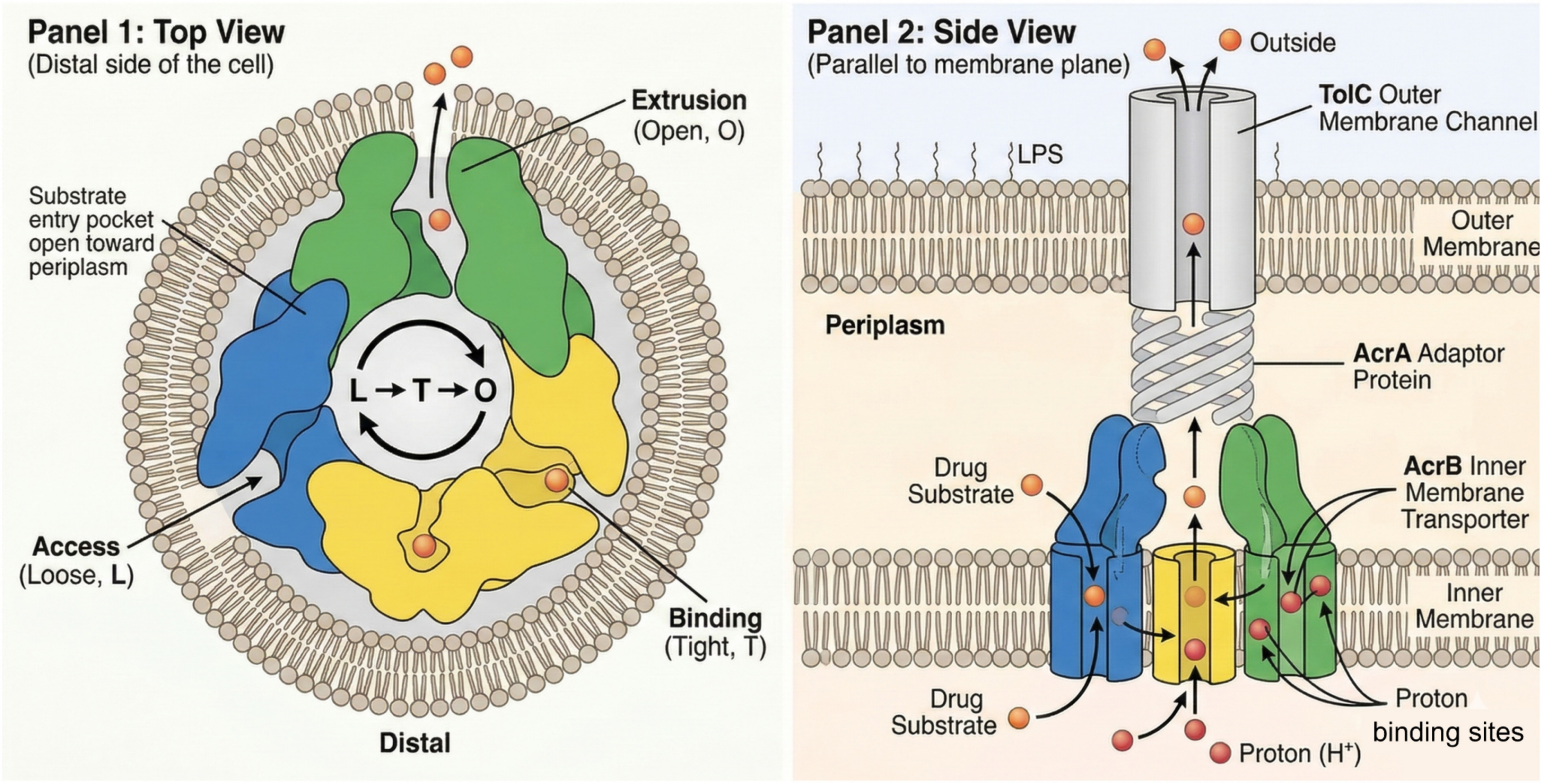
Schematic representation of the AcrAB-TolC multidrug efflux pump in Gram-negative bacteria. Panel 1 (Top view) shows the AcrB trimer cycling through three conformational states—Access (Loose, L), Binding (Tight, T), and Extrusion (Open, O)—that enable sequential substrate uptake, binding, and export. Panel 2 (Side view) illustrates the tripartite AcrAB-TolC complex spanning the inner membrane, periplasm, and outer membrane, where AcrB uses the proton motive force to transport drug substrates through AcrA to the TolC channel for extrusion outside the cell.

Experimental and computational studies have increasingly elucidated how membrane potential dynamics regulate bacterial efflux pumps, particularly those of the resistance-nodulation-division (RND) family, such as AcrB. Efflux pumps harness the proton motive force (PMF), an electrochemical gradient across the inner membrane, as the primary energy source for substrate extrusion. Disruption or alteration of this membrane potential directly affects pump activity and efficiency (Whittle et al., 2024). Genetic knockout or functional inactivation of efflux pumps leads to increased bacterial membrane potential (hyperpolarization) due to decreased proton influx in bacterial cells. In the previous literature it has been demonstrated that *Salmonella* strains deficient in the AcrB pump or carrying mutations that block proton translocation exhibit significantly elevated membrane potential relative to wild-type cells (Whittle et al., 2024). This hyperpolarization affects global cellular physiology, including shifts in anaerobic energy metabolism, redox balance, and delayed entry into the stationary phase, establishing a direct link between efflux function and membrane energetics (Whittle et al., 2024). The protonophore CCCP collapse of PMF is routinely used experimentally to confirm the dependence of efflux on membrane potential (López et al., 2017). At the computational level, molecular dynamics (MD) simulations of intact tripartite pumps embedded in Gram-negative inner and outer membrane models reveal how pump conformations and inter-protein interfaces are energetically coupled to the surrounding membrane environment and proton gradients (López et al., 2017). MD studies of MexAB-OprM from *Pseudomonas aeruginosa* reveal that the conformational dynamics responsible for opening the outer membrane channel and substrate extrusion are intimately regulated by proton flux-driven changes in the transmembrane regions, which are sensitive to variations in membrane potential (López et al., 2017). Simulations also clarify how allosteric inhibitor binding within transmembrane pockets interfere with proton relay and membrane-driven conformational cycling, offering molecular insight into efflux modulation (Plé et al., 2022; Roy et al., 2024). MD simulations predicted pyridylpiperazine inhibitors to preferentially interact with the cytosolic surface of the L protomer. Specifically, simulations indicated that compounds enter AcrB in the L protomer state via a cytoplasmic-open access channel leading to the inhibitor binding pocket (Figure 4; Plé et al., 2022). As the L protomer TM domain is in an inward open conformation, this entry pathway is more accessible than in the T or the O protomer, which are in an outward open (inward closed) or occluded conformation, respectively (Eicher et al., 2014).

### 12.4 Impact of membrane potential perturbation on efflux efficiency and drug accumulation

The efficiency of bacterial efflux pumps is closely linked to the energetic state of the cell, particularly the membrane potential (ΔΨ) component of the proton motive force (PMF), which drives proton-dependent transport systems, including the RND, MFS, and MATE pump families. Perturbation of ΔΨ reduces the energetic coupling required for substrate export, leading to impaired efflux and increased intracellular retention of antibiotics. For example, the artificial dissipation of ΔΨ using protonophores, such as CCCP, significantly increases intracellular drug accumulation in *Escherichia coli* and *Salmonella enterica*, demonstrating that efflux pump activity is directly dependent on maintaining membrane electrochemical gradients (Wang et al., 2021). Recent findings further reveal a bidirectional relationship in which loss or inhibition of major efflux systems, such as AcrB, alters membrane potential itself, indicating that ΔΨ not only drives but is also stabilized by efflux function (Whittle et al., 2024). Additionally, membrane potential influences the translocation of charged antibiotics across the membrane, affecting both uptake and export kinetics (Hillman, 2023). Computational analysis also supports the concept that PMF modulation can tune efflux specificity and turnover efficiency (Gerry et al., 2025). Together, these observations highlight ΔΨ as a critical regulatory axis in antimicrobial resistance, suggesting that targeted interference with membrane energetics may serve as a promising strategy to enhance intracellular drug accumulation and restore antibiotic susceptibility.

### 12.5 Influence of bacterial pH, nutrient availability, and pharmacokinetic considerations on allosteric efflux pump inhibitors

The activity and accessibility of allosteric efflux pump inhibitors (EPIs) are strongly influenced by the physiological microenvironment of bacterial cells, including variations in pH, nutrient availability, and metabolic state. Changes in environmental pH can significantly modulate the proton motive force (PMF), depends on the membrane potential (ΔΨ) and the transmembrane proton gradient (ΔpH), both of which drive the catalytic cycle of proton-dependent efflux pumps such as AcrAB–TolC. Changes in extracellular or cytoplasmic pH may therefore affect protonation states of both efflux pump residues and inhibitor molecules, potentially influencing the accessibility, binding affinity, and stability of allosteric inhibitor interactions within the transmembrane regulatory pocket of AcrB (Plé et al., 2022; Yang et al., 2023). Environmental pH changes can affect membrane permeability and the electrochemical gradient (membrane potential) across the bacterial membrane, affecting the diffusion or accumulation of EPIs in the periplasmic space, where many regulatory binding sites are located (Hillman, 2023; Wan et al., 2023). Under nutrient-rich conditions, metabolism sustains strong PMF generation through the electron transport chain, enabling robust efflux activity. In contrast, nutrient limitation or stationary-phase growth can lead to metabolic remodeling and adaptive regulation of PMF to maintain essential processes, including multidrug efflux, despite reduced energy availability (Wang et al., 2021; Wan et al., 2023). Such metabolic adaptations may influence the structural accessibility of allosteric binding pockets, thereby affecting the inhibitory efficiency of EPIs. Many promising inhibitors exhibit potent *in-vitro* activity but face significant barriers related to absorption, distribution, metabolism, and excretion (ADME), as well as limited penetration across the Gram-negative outer membrane (Thakur et al., 2021; Duffey et al., 2024). Determining the dissociation constants (KD) and binding free energies of allosteric inhibitors across different environmental pH ranges would provide critical insights into how protonation states of efflux pump residues and inhibitor molecules influence binding affinity and residence time at the allosteric regulatory site. Acidic environmental conditions have been reported to stimulate transcriptional upregulation of multidrug efflux systems in several Gram-negative bacteria, which may further reduce intracellular accumulation of EPIs and compromise their inhibitory activity. Therefore, systematic biophysical and biochemical studies measuring pH-dependent KD values and inhibitor residence times will be essential to clarify how bacterial microenvironmental factors influence allosteric inhibitor binding and to guide the rational design of next-generation efflux pump inhibitors (Nikaido and Pagès, 2012; Li et al., 2015; Wang et al., 2017; Du et al., 2018).

## 13 Synergy in combination therapy: antibiotic potentiation, resistance prevention, and *in-vivo* efficacy

Drug combinations can amplify antibacterial activity by targeting complementary pathways, improving cell entry, or disabling resistance mechanisms, thereby potentiating “old” drugs and expanding therapeutic windows. Mechanistically informed pairings—such as membrane permeabilizers with hydrophilic antibiotics, β-lactams with β-lactamase inhibitors, or efflux pump inhibitors (EPIs) with pump substrates—have repeatedly demonstrated supra-additive killing *in-vitro* and translational promise *in-vivo* (Bognár et al., 2024; Garza-Cervantes and León-Buitimea, 2024). A central objective of combination therapy is resistance prevention—slowing de novo resistance and suppressing pre-existing resistant subpopulations. Beyond antibiotics, phage–antibiotic combinations can deliver potent synergism and reduced resistance emergence when partners are orthogonal in mechanism—e.g., cell-wall active antibiotics with lytic phages—expanding the design space for anti-resistance therapy (Manohar et al., 2022). Recent state-of-the-art surveys have cataloged successful AMP–antibiotic and antibiotic–adjuvant combinations that reduce bacterial loads and improve outcomes *in-vivo*, reinforcing the translational potential of synergy when guided by mechanism (Garza-Cervantes and León-Buitimea, 2024). Together, these advances support mechanism-driven, pharmacodynamically tuned combination regimens that potentiate index drugs through permeabilization, efflux inhibition, enzyme neutralization, and resistance prevention, and are increasingly reflected in contemporary drug development pipelines and clinical trial designs (Si et al., 2023; Bognár et al., 2024).

## 14 Diagnostic and translational perspectives

Recent advances in phenotypic diagnostics have enabled rapid quantification of efflux-pump activity and membrane potential in clinical bacterial isolates—tools that are critical for translating mechanistic resistance insights into actionable diagnostics. Fluorescent dye-based efflux assays, such as Nile Red or ethidium bromide accumulation, when coupled with flow cytometry or microplate fluorimetry, enable real-time assessment of efflux capacity in clinical strains of *Enterobacterales* and *Staphylococcus aureus*, thereby correlating efflux kinetics with antimicrobial susceptibility phenotypes (Whittle et al., 2024). Parallel advances in membrane potential probes [(e.g., DiOC2 (Mascellino et al., 2024), DiSC3 (Gaurav et al., 2023)] enable the quantification of ΔΨ in living bacteria, allowing for the stratification of isolates by energetic phenotype and linking membrane hyperpolarization or depolarization with drug tolerance or efflux competence (Zand et al., 2021). Microfluidic single-cell platforms further promise ultra-rapid (<1 h) readouts of membrane potential shifts under antibiotic challenge, offering potential for point-of-care diagnostics of efflux-mediated resistance (Zagajewski et al., 2023). Collectively, the convergence of efflux and membrane potential assays provides a translational pipeline through which laboratory mechanistic findings can inform clinical decisions—such as identifying high-efflux phenotypes that may benefit from efflux-pump inhibitors or membrane-disrupting adjuvants in real time.

## 15 Validation and clinical outlooks for allosteric inhibitors and PMF-targeting agents

Several recent investigations highlight the translational promise of both allosteric inhibitors and proton-motive-force (PMF)-targeting agents for combating antimicrobial-resistant infections *in-vivo*. These findings are primarily derived from well-established animal models, including murine systemic infection models, pulmonary infection models, and MRSA infection models, which enable assessment of bacterial burden, survival outcomes, and therapeutic efficacy (Hosfelt et al., 2022). On the bioenergetics front, detailed reviews show that disruption of bacterial PMF—encompassing ΔΨ and ΔpH—undermines key physiological processes including ATP synthesis and efflux pump activity, thereby sensitizing pathogens in animal infection models and suggesting a clinically relevant paradigm for adjuvant therapy (Yang et al., 2023). Moreover, experimental work that applied small-molecule PMF dissipators to methicillin-resistant *Staphylococcus aureus* demonstrated potent bactericidal activity *in-vivo* and synergistic combination with conventional antibiotics (Mohiuddin et al., 2022). Together, the *in-vivo* data for both modalities reinforce the concept that mechanistically orthogonal interventions—targeting bacterial energetics or non-active-site binding—can expand the therapeutic toolkit, reduce the probability of cross-resistance, and provide viable leads for clinical development of next-generation anti-infectives.

## 16 Challenges and future directions in overcoming resistance to allosteric inhibitors

Allosteric inhibitors have emerged as a promising class of therapeutics capable of modulating protein function by binding to regulatory sites distinct from the orthosteric (active) site. Their unique mechanisms provide advantages in overcoming resistance mutations that typically affect orthosteric inhibitors. However, resistance to allosteric inhibitors themselves presents a critical challenge to their long-term clinical efficacy. One significant challenge is the emergence of resistance mutations within the allosteric binding pockets or along the allosteric communication pathways. Such mutations can reduce the inhibitor’s binding affinity or impair the allosteric modulation of the target, thereby diminishing the drug’s potency (Lu et al., 2020). In addition, up-regulation of efflux transporters can lower intracellular concentrations of allosteric inhibitors, thereby compromising efficacy. Future strategies to overcome these barriers involve the rational design of next-generation allosteric inhibitors with enhanced binding specificity and resistance-resilient mechanisms. Combination therapies employing both orthosteric and allosteric inhibitors—either as separate agents or linked “bitopic” molecules—offer a promising avenue for reducing resistance development by simultaneously targeting multiple regulatory sites (Zhang et al., 2022). Advances in computational modeling and structural biology enable the prediction and optimization of allosteric sites and their dynamic properties, facilitating the design of inhibitors less prone to resistance mutations (Ni et al., 2020; Outhwaite et al., 2025).

## 17 Future research gaps and methodological needs

Despite significant advances, critical research gaps and methodological challenges persist that hamper progress in many biomedical fields, including antibiotic discovery, drug delivery, and resistance mitigation. One major gap is the insufficient integration of interdisciplinary approaches, which limits a holistic understanding of complex physiological processes and bacterial resistance mechanisms. Future research must prioritize comprehensive frameworks that encompass molecular, cellular, and systemic levels to overcome these limitations (Hamers et al., 2023). Methodological deficiencies also impede the reproducibility and robustness of findings. Outdated or inadequate experimental models, lack of standardized protocols, and insufficiently representative preclinical systems constrain translation. Sophisticated, physiologically relevant models—such as organoids, microfluidic systems, and advanced *in-vivo* imaging—are urgently needed to better simulate human pathophysiology and drug interactions (Jansson-Löfmark et al., 2025). Data-related challenges include heterogeneity, poor integration across datasets, and unresolved ethical concerns regarding data privacy, particularly in clinical research. Developing standardized, interoperable data platforms and applying advanced analytics like artificial intelligence will enhance data interpretability and accelerate discovery. However, these tools require careful validation and transparency to avoid bias and ensure reliability (Nyanchoka et al., 2019). There remains a pressing need for methodological innovation in clinical trial designs, particularly in adaptive and pragmatic trials that better reflect real-world settings and facilitate the rapid and efficient evaluation of novel interventions. Addressing gaps in patient diversity, including underrepresented populations and variable disease phenotypes, will further strengthen evidence and equity in healthcare outcomes (Hamers et al., 2023).

## 18 Conclusion

Next-generation antimicrobial therapies have witnessed significant advances through the discovery of novel antimicrobial compounds, innovative drug delivery platforms, and alternative therapeutic modalities, including bacteriophage therapy, antimicrobial peptides, and efflux pump inhibitors. These developments hold promise for overcoming the current limitations posed by multidrug-resistant pathogens and reinvigorating the antimicrobial pipeline. Comprehensive surveillance data emphasize the critical need for integrating these novel approaches with global stewardship and rapid diagnostics to enhance clinical impact (Naghavi et al., 2024). Looking forward, next-generation antimicrobial strategies must embrace precision medicine frameworks, leveraging omics technologies and artificial intelligence to tailor therapies to pathogen and patient-specific profiles. Enhanced global cooperation for data sharing, harmonized surveillance, and sustainable financing models will be essential to accelerate innovation and equitable deployment. Ultimately, a holistic and coordinated effort that bridges scientific innovation with public health imperatives will be crucial for mitigating the threat of antimicrobial resistance and ensuring effective infection management in the decades to come (Huang et al., 2025).
